# The In Vitro Effect of Prostaglandin E_2_ and F_2α_ on the Chemerin System in the Porcine Endometrium during Gestation

**DOI:** 10.3390/ijms21155213

**Published:** 2020-07-23

**Authors:** Kamil Dobrzyn, Marta Kiezun, Ewa Zaobidna, Katarzyna Kisielewska, Edyta Rytelewska, Marlena Gudelska, Grzegorz Kopij, Kinga Bors, Karolina Szymanska, Barbara Kaminska, Tadeusz Kaminski, Nina Smolinska

**Affiliations:** Department of Animal Anatomy and Physiology, Faculty of Biology and Biotechnology, University of Warmia and Mazury in Olsztyn, Oczapowskiego 1A, 10-719 Olsztyn-Kortowo, Poland; marta.kiezun@uwm.edu.pl (M.K.); ewa.zaobidna@uwm.edu.pl (E.Z.); katarzyna.kisielewska@uwm.edu.pl (K.K.); edyta.rytelewska@uwm.edu.pl (E.R.); marlena.gudelska@uwm.edu.pl (M.G.); grzegorz.kopij@uwm.edu.pl (G.K.); kinga.bors@uwm.edu.pl (K.B.); karolina.szymanska@uwm.edu.pl (K.S.); barbara.kaminska@uwm.edu.pl (B.K.); tkam@uwm.edu.pl (T.K.)

**Keywords:** early pregnancy, chemerin, chemerin receptors, porcine uterus, PGE_2_, PGF_2α_

## Abstract

Chemerin belongs to the group of adipocyte-derived hormones known as adipokines, which are responsible mainly for the control of energy homeostasis. Adipokine exerts its influence through three receptors: Chemokine-like receptor 1 (CMKLR1), G protein-coupled receptor 1 (GPR1), and C-C motif chemokine receptor-like 2 (CCRL2). A growing body of evidence indicates that chemerin participates in the regulation of the female reproductive system. According to the literature, the expression of chemerin and its receptors in reproductive structures depends on the local hormonal milieu. The aim of this study was to investigate the in vitro effect of prostaglandins E_2_ (PGE_2_) and F_2α_ (PGF_2α_) on chemerin and chemerin receptor (chemerin system) mRNAs (qPCR) and proteins (ELISA, Western blotting) in endometrial tissue explants collected from early-pregnant gilts. Both PGE_2_ and PGF_2α_ significantly influenced the expression of the chemerin gene, hormone secretion, and the expression of chemerin receptor genes and proteins. The influence of both prostaglandins on the expression of the chemerin system varied between different stages of gestation. This is the first study to describe the modulatory effect of PGE_2_ and PGF_2α_ on the expression of the chemerin system in the porcine uterus during early gestation.

## 1. Introduction

The relationship between nutritional status and reproductive success in animals has been studied for many years. Adipokines are a group of adipocyte-derived agents that are responsible mainly for the regulation of energy metabolism, and they are perceived as the key link between metabolic status and reproductive functions. The adipokine family includes chemerin, the product of the retinoic acid receptor responder 2 gene (*RARRES2*), also known as tazarotene-induced gene 2 (*TIG2*) [1]. Chemerin circulates in the blood as prochemerin, which consists of 143 amino acids and originates from a 163 amino acid precursor known as pre-prochemerin [2]. Interestingly, chemerin has been found to exert pleiotropic effects, including modulation of insulin sensitivity that affects food intake, energy homeostasis, and adipose tissue function [3,4]. Chemerin has been found to have a chemotactic properties for immune cells, through promoting the immune cells migration to the inflammation sites. It has also been reported that chemerin may inhibit pro-inflammatory cytokines actions, such as interleukin-6 (IL-6) and tumor necrosis factor-α (TNF-α) [3,5]. In the body, chemerin binds to three G-protein coupled receptors: Chemokine-like receptor 1 (CMKLR1), G protein-coupled receptor 1 (GPR1), and C-C motif chemokine receptor-like 2 (CCRL2). Chemokine-like receptor 1, also known as ChemR23 in humans, has been most extensively studied. G protein-coupled receptor 1 is structurally similar to CMKLR1, but its role has not been elucidated to date. It is believed that GPR1 has different functions than those of CMKLR1 because both receptors are expressed in different tissues. Chemokine-like receptor 1 has been detected mainly in macrophages, natural killer cells, plasmacytoid dendritic cells, and myeloid dendritic cells, whereas GPR1 is expressed in cells related to the central nervous system [6,7,8]. CMKLR1 has been found to use MAPK/ERK1/2, AMPK, and PI3K/Akt kinases to transduce signals, whereas GPR1 acts mainly through the AMPK signaling pathway [9]. The third receptor, CCRL2, differs from CMKLR1 and GPR1. C-C motif chemokine receptor-like 2 is unable to transduce the signal, but it binds the N-terminal region of chemerin and exposes the C-terminal region of the hormone molecule to CMKLR1 that is expressed on the membranes of the neighboring cells [10]. The expression of the CCLR2 gene and protein was confirmed in neutrophils, T cells, and macrophages [11].

A growing body of evidence suggests that chemerin may be responsible for the control of reproductive functions. It has been indicated that the inhibition of the chemerin signaling via the CMKLR1 receptor in the mice decidua results in embryo abortion [12]. Furthermore, it has been observed that in women who had early spontaneous abortions, the concentrations of chemerin in the blood plasma were decreased, whereas the expression of CMKLR1 in the decidua was found to be elevated [12]. The expression of the chemerin system (a collective term for chemerin and its receptors) was confirmed in brain structures responsible for reproductive functions, as well as in the lower branches of the hypothalamic-pituitary-ovarian axis and the female reproductive tract. Chemerin expression was confirmed in the hypothalami of mice and pigs [13,14]. Chemerin receptor expression was reported in porcine and bovine pituitaries and hypothalami [14,15,16]. It has been suggested that chemerin plays an important role in the structural remodeling of the hypothalamus, and may be responsible for the regulation of the secretion of hypothalamic hormones connected with feeding behavior [13,17]. The expression of chemerin and its receptors in the porcine hypothalamic structures responsible for gonadoliberin (GnRH) production, as well as in the pituitary gonadotrophs suggests the potential involvement of the adipokine in the regulation of the reproductive functions [14,15]. Chemerin system expression was observed in the human uterus [18,19], in human and rat placenta [1,20], in the ovaries of women and rodents [1,21,22], and in porcine ovaries, uteri, trophoblasts, and conceptuses [23,24]. The expression of the chemerin system components in the endometrium, trophoblasts, placentas, and conceptuses suggests that the adipokine may play an important role in the foeto–maternal crosstalk during early pregnancy, as well as, through the placenta, across the whole gestation period. Chemerin decreases the in vitro steroidogenesis of granulosa cells, blocks oocyte meiotic progression in cattle, and suppresses FSH-induced progesterone (P_4_) and estradiol (E_2_) secretion in rat preantral follicles and granulosa cells [21,22,25]. Chemerin also reduced the hCG-induced production of P_4_ in cultured follicles and corpora lutea of mice [26]. Our previous research revealed changes in the expression of the chemerin system in porcine uterine and ovarian tissues and demonstrated changes in serum chemerin concentrations during the estrous cycle and early gestation. These findings suggest that chemerin system expression may be dependent on the hormonal milieu [14,23,24]. Similar observations were made by Yang et al., [26] who reported that chemerin and GPR1 expression in the ovaries of mice varied during the estrous cycle. Treatment involving PGF_2α_ decreased GPR1 receptor expression at both gene and protein levels. The above findings supported the formulation of the research hypothesis postulating that prostaglandins, the key factors that regulate uterine functions in early pregnancy, affect the endometrial expression of the chemerin system. Therefore, the aim of the present study was to investigate the influence of prostaglandin E_2_ (PGE_2_) and F_2α_ (PGF_2α_) on the endometrial expression of chemerin, *CMKLR1*, *GPR1,* and *CCRL2* genes, and on the concentrations of chemerin receptor proteins and chemerin secretion by endometrial tissue explants during early gestation.

## 2. Results

### 2.1. The Effect of PGE_2_ on Chemerin Gene Expression and Protein Secretion by the Endometrial Tissue Explants

**Figure 1:**
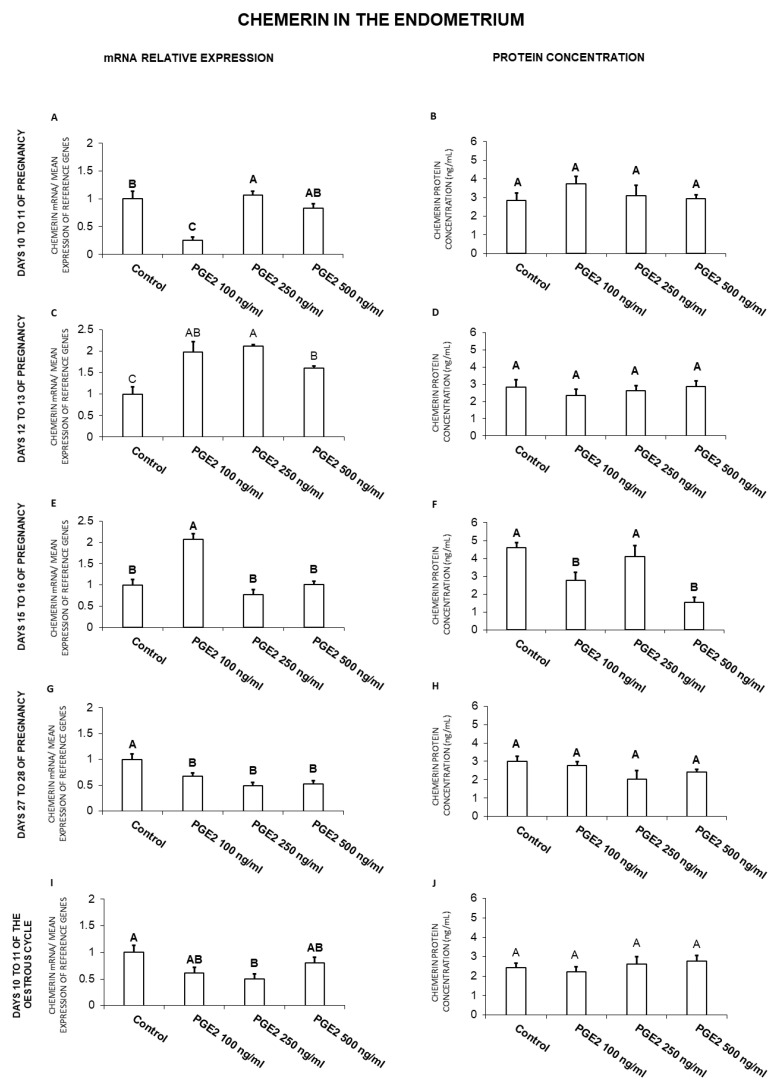
The influence of prostaglandin E_2_ (PGE_2_; 100, 250, 500 ng/mL) on chemerin mRNA expression (**A**,**C**,**E**,**G**,**I**) and chemerin protein secretion (**B**,**D**,**F**,**H**,**J**) in the porcine endometrial tissue explants on days 10 to 11, 12 to 13, 15 to 16 and 27 to 28 of the pregnancy, and on days 10 to 11 of the estrous cycle. The gene expression was determined by quantitative real-time PCR. The protein secretion was determined by an ELISA test. Results are reported as the means ± S.E.M. (*n* = 5). Bars with different superscripts differ (*p* < 0.05).

In the endometrium, on days 10 to 11 of gestation, PGE_2_ at the dose of 250 ng/mL enhanced, but at the dose of 100 ng/mL decreased chemerin gene expression (Figure 1A). On days 12 to 13 of gestation, PGE_2_ at all the tested doses caused an increase in chemerin mRNA content (Figure 1C). On days 15 to 16 of pregnancy, PGE_2_ enhanced chemerin gene expression (100 ng/mL), but decreased the hormone secretion (100 and 500 ng/mL; Figure 1E,F). Prostaglandin E_2_ suppressed the endometrial chemerin gene expression on days 27 to 28 of pregnancy (100, 250, 500 ng/mL), as well as on days 10 to 11 of the estrous cycle (250 ng/mL; Figure 1G,I; *p* < 0.05).

In general, PGE_2_ treatment resulted in an increase of chemerin mRNA expression on days 12 to 13 and 15 to 16 of pregnancy and a decrease on days 27 to 28 of gestation and days 10 to 11 of the cycle. PGE_2_ decreased chemerin protein secretion on days 15 to 16 of gestation.

### 2.2. The Effect of PGE_2_ on CMKLR1 Gene and Protein Expression in the Endometrial Tissue Explants

**Figure 2:**
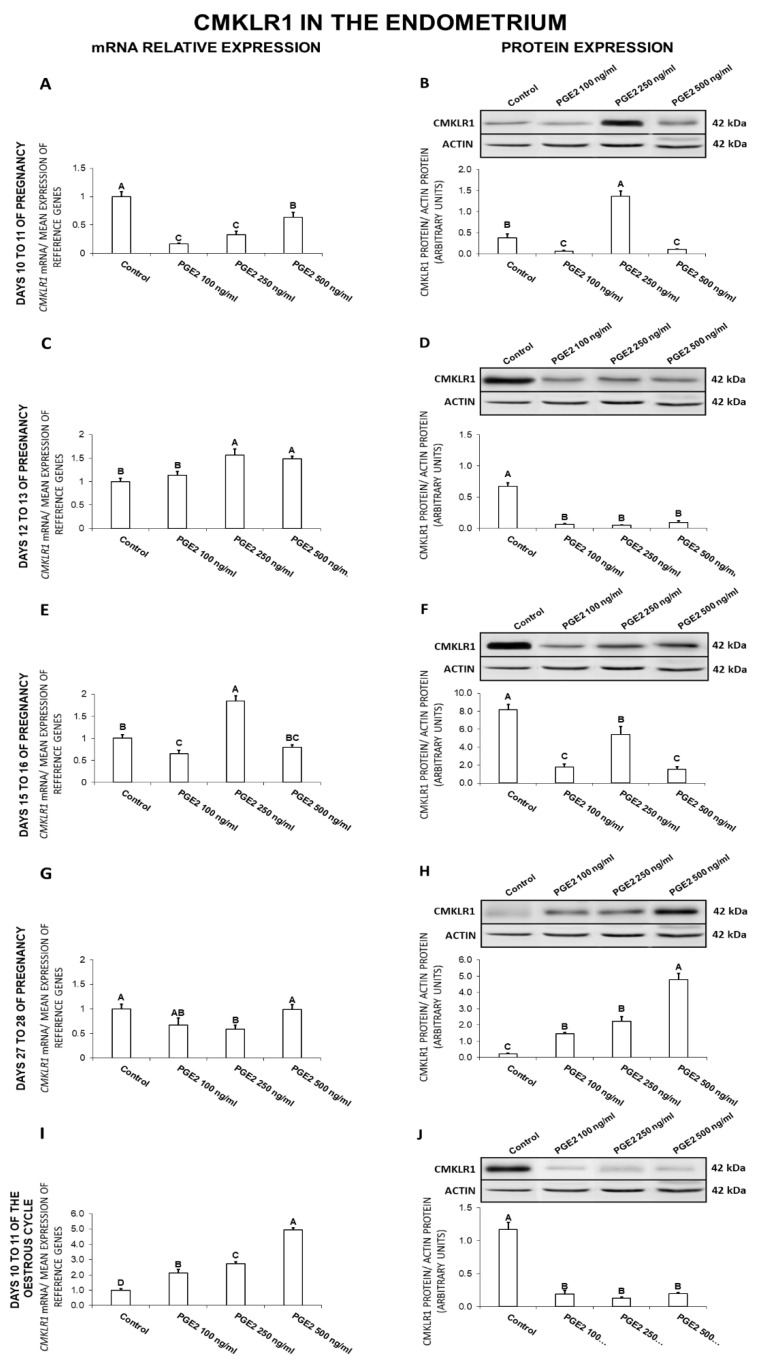
The influence of prostaglandin E_2_ (PGE_2_; 100, 250, 500 ng/mL) on chemokine-like receptor 1 (CMKLR1) mRNA (**A**,**C**,**E**,**G**,**I**) and protein (**B**,**D**,**F**,**H**,**J**) expression in the porcine endometrium on days 10 to 11, 12 to 13, 15 to 16, and 27 to 28 of the pregnancy, and on days 10 to 11 of the estrous cycle. The gene expression was determined by quantitative real-time PCR. The protein concentration was determined by the western blotting analysis; upper panels: Representative immunoblots; lower panels: Densitometric analysis of CMKLR1 protein relative to actin protein. Results are reported as the means ± S.E.M. (*n* = 5). Bars with different superscripts differ (*p* < 0.05).

In the endometrium, on days 10 to 11 of gestation, PGE_2_ (100, 250, 500 ng/mL) caused a decrease in *CMKLR1* gene expression (Figure 2A). During the same days, PGE_2_ at the dose of 250 ng/mL increased, whereas at doses of 100 and 500 ng/mL decreased the receptor protein concentration (Figure 2B). On days 12 to 13 of pregnancy, PGE_2_ increased (250, 500 ng/mL) *CMKLR1* mRNA content and decreased (100, 250, 500 ng/mL) the receptor protein concentration (Figure 2C,D). On days 15 to 16 of gestation, PGE_2_ at the dose of 250 ng/mL enhanced, but at the dose of 100 ng/mL suppressed *CMKLR1* mRNA content (Figure 2E). During the same days, PGE_2_ caused a decrease in the receptor protein concentration (100, 250, 500 ng/mL; Figure 2F). On days 27 to 28 of pregnancy, PGE_2_ inhibited *CMKLR1* gene expression (250 ng/mL) and enhanced the receptor protein concentration (100, 250, 500 ng/mL; Figure 2G,H). On days 10 to 11 of the estrous cycle, PGE_2_ at all tested doses caused an increase in *CMKLR1* gene expression but decreased the receptor protein concentration (Figure 2J; *p* < 0.05).

In general, PGE_2_ decreased *CMKLR1* gene expression on days 10 to 11 and 27 to 28 of gestation and protein expression on days 12 to 13 and 15 to 16 of gestation, and 10 to 11 of the cycle. PGE_2_ enhanced the receptor gene expression on days 12 to 13 of gestation and 10 to 11 of the cycle, and increased CMKLR1 protein content on days 27 to 28 of pregnancy.

### 2.3. The Effect of PGE_2_ on GPR1 Gene and Protein Expression in the Endometrial Tissue Explants

**Figure 3:**
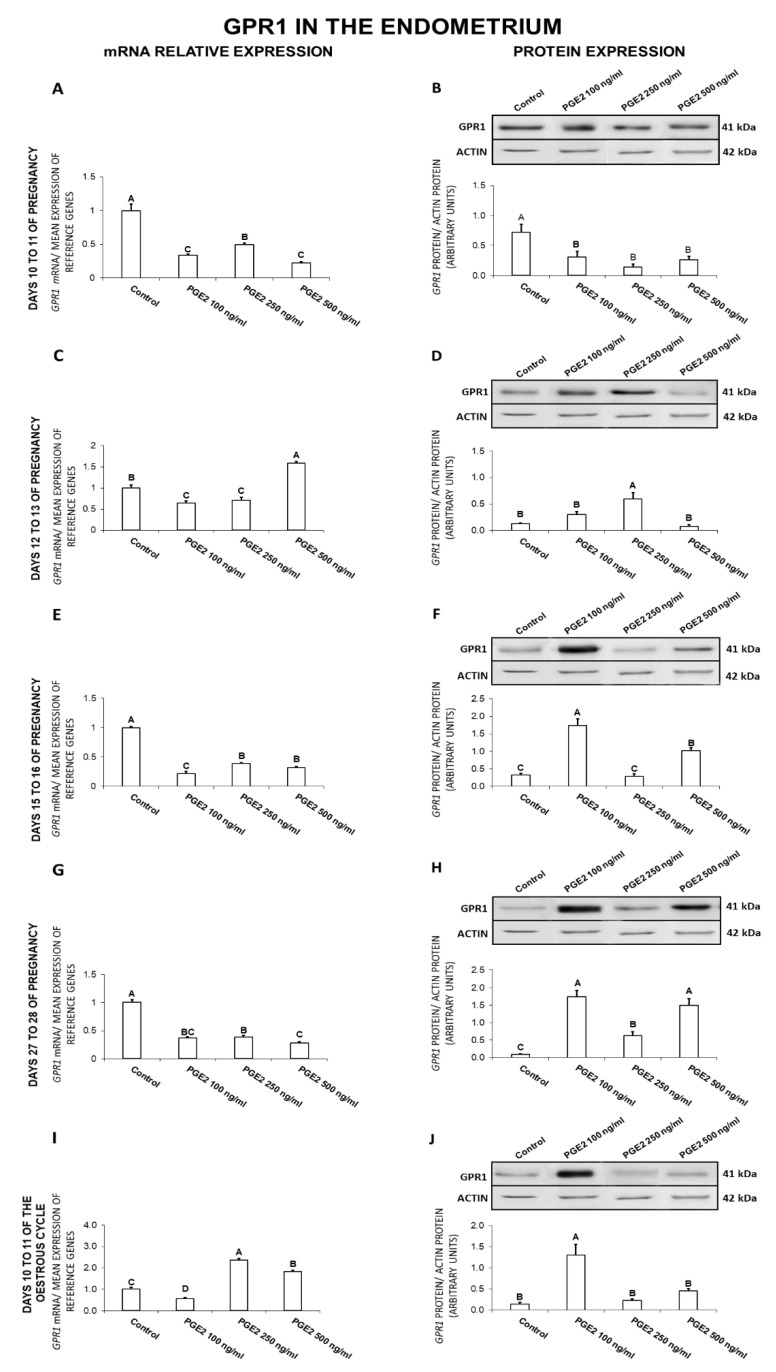
The influence of prostaglandin E_2_ (PGE_2_; 100, 250, 500 ng/mL) on G protein-coupled receptor 1 (GPR1) mRNA (**A**,**C**,**E**,**G**,**I**) and protein (**B**,**D**,**F**,**H**,**J**) expression in the porcine endometrium on days 10 to 11, 12 to 13, 15 to 16, and 27 to 28 of the pregnancy, and on days 10 to 11 of the estrous cycle. The gene expression was determined by quantitative real-time PCR. The protein concentration was determined by the western blotting analysis; upper panels: Representative immunoblots; lower panels: Densitometric analysis of GPR1 protein relative to actin protein. Results are reported as the means ± S.E.M. (*n* = 5). Bars with different superscripts differ (*p* < 0.05).

On days 10 to 11 of gestation, PGE_2_ at all tested doses caused a decrease in GPR1 expression at both the gene and protein levels (Figure 3A,B). On days 12 to 13 of gestation, PGE_2_ at the dose of 500 ng/mL increased, but at the doses of 100 and 250 ng/mL inhibited *GPR1* gene expression. During the same days, PGE_2_ (250 ng/mL) caused an increase in the endometrial GPR1 protein concentration (Figure 3C,D). On days 15 to 16 of pregnancy, PGE_2_ caused a decrease in *GPR1* mRNA content (100, 250, 500 ng/mL) and increased the receptor protein concentration (100, 500 ng/mL; Figure 3E,F). On days 27 to 28 of pregnancy, PGE_2_ at all the tested doses caused a decrease in the receptor gene expression and provoked an increase in GPR1 protein concentration (Figure 3G,H). On days 10 to 11 of the estrous cycle, PGE_2_ at the doses of 250 and 500 ng/mL enhanced, but at the dose of 100 ng/mL inhibited the endometrial *GPR1* expression. During the same period, PGE_2_ at the dose of 100 ng/mL was found to increase the receptor protein concentration (Figure 3I,J; *p* < 0.05).

Generally, PGE_2_ decreased the receptor mRNA content on days 10 to 11, 15 to 16, and 27 to 28 of pregnancy and GPR1 protein concentration on days 10 to 11 of pregnancy. The prostaglandin increased GPR1 protein concentration on days 12 to 13, 15 to 16, and 27 to 28 of pregnancy, and 10 to 11 of the cycle.

### 2.4. The Effect of PGE_2_ on CCRL2 Gene and Protein Expression in the Endometrial Tissue Explants

**Figure 4:**
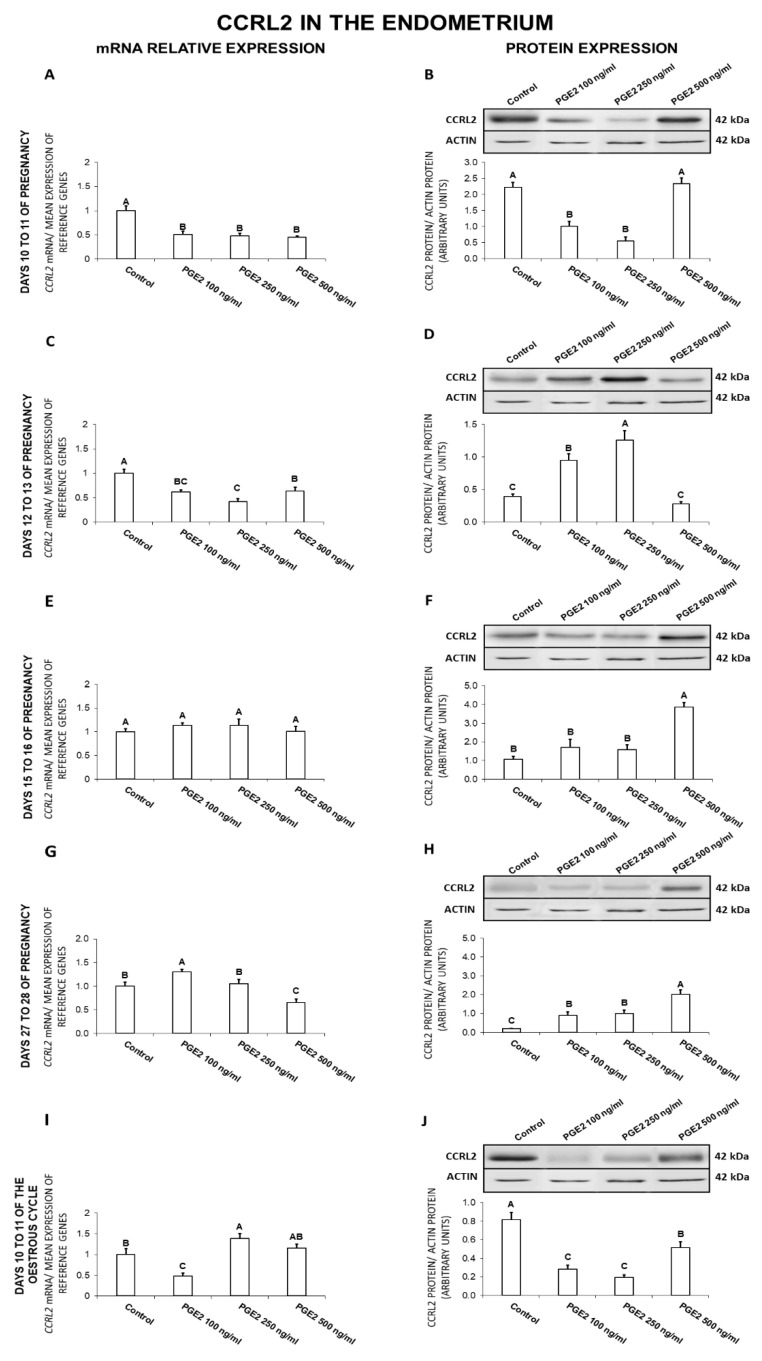
The influence of prostaglandin E_2_ (PGE_2_; 100, 250, 500 ng/mL) on C-C motif chemokine receptor like 2 (CCRL2) mRNA (**A**,**C**,**E**,**G**,**I**) and protein (**B**,**D**,**F**,**H**,**J**) expression in the porcine endometrium on days 10 to 11, 12 to 13, 15 to 16, and 27 to 28 of the pregnancy, and on days 10 to 11 of the estrous cycle. The gene expression was determined by quantitative real-time PCR. The protein concentration was determined by the western blotting analysis; upper panels: Representative immunoblots; lower panels: Densitometric analysis of CCRL2 protein relative to actin protein. Results are reported as the means ± S.E.M. (*n* = 5). Bars with different superscripts differ (*p* < 0.05).

On days 10 to 11 of gestation, PGE_2_ was found to exert an inhibitory effect on the endometrial expression of CCRL2 at both the gene (100, 250, 500 ng/mL) and protein (100, 250 ng/mL) levels (Figure 4A,B). On days 12 to 13 of gestation, PGE_2_ caused a decrease (100, 250, 500 ng/mL) in *CCRL2* expression, but increased (100, 250 ng/mL) the receptor protein concentration (Figure 4C,D). On days 15 to 16 of pregnancy, PGE_2_ at the dose of 500 ng/mL caused an increase in CCRL2 protein concentration (Figure 4F). On days 27 to 28 of pregnancy, PGE_2_ at the dose of 100 ng/mL increased, whereas at the dose of 500 ng/mL decreased *CCRL2* mRNA content (Figure 4G). On these days, PGE_2_ at all the tested doses caused an increase in the endometrial CCRL2 protein concentration (Figure 4H). On days 10 to 11 of the estrous cycle, PGE_2_ at the dose of 250 ng/mL enhanced, but at the dose of 100 ng/mL inhibited *CCRL2* expression (Figure 4I). On these days, PGE_2_ was observed to decrease (100, 250, 500 ng/mL) the endometrial CCRL2 protein concentration (Figure 4J; *p* < 0.05).

In summary, PGE_2_ inhibited CCRL2 gene expression on days 10 to 11 and 12 to 13 of gestation, and the receptor protein content on days 10 to 11 of pregnancy and 10 to 11 of the cycle. On days 12 to 13, 15 to 16, and 27 to 28, PGE_2_ stimulated CCRL2 protein expression.

### 2.5. The Effect of PGF_2α_ on Chemerin Gene Expression and Protein Secretion by the Endometrial Tissue Explants

**Figure 5:**
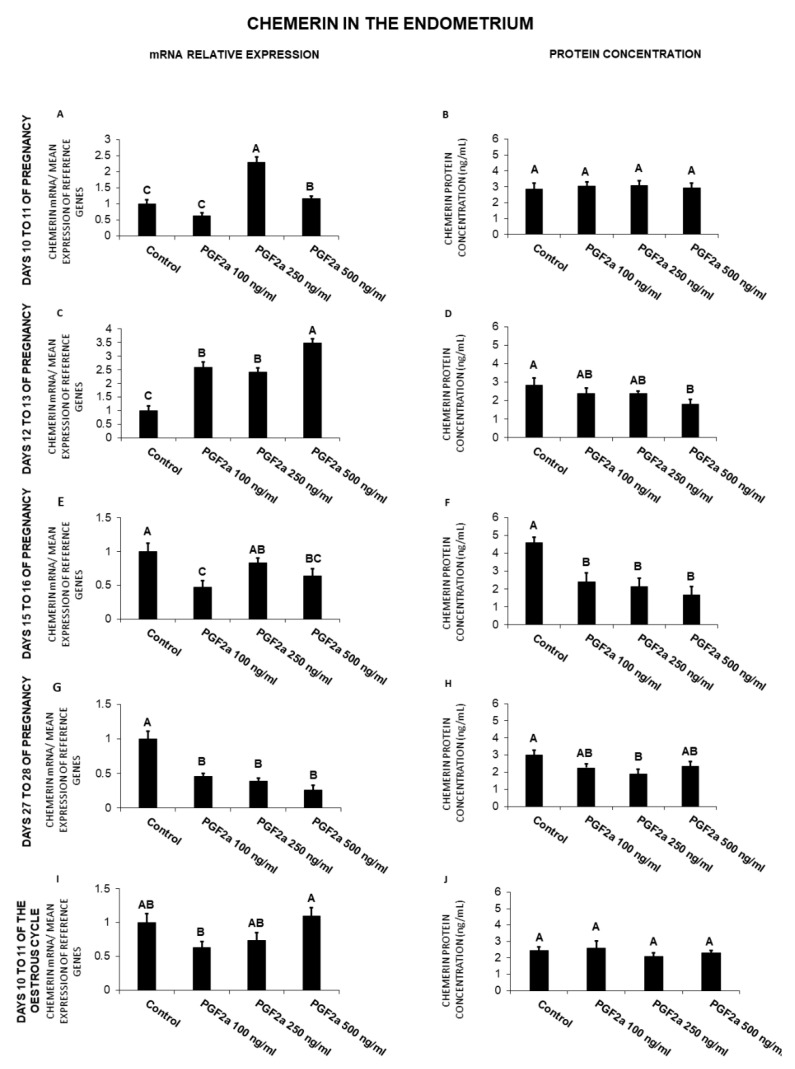
The influence of prostaglandin F_2α_ (PGF_2α_; 100, 250, 500 ng/mL) on chemerin mRNA expression (**A**,**C**,**E**,**G**,**I**) and chemerin protein secretion (**B**,**D**,**F**,**H**,**J**) in the porcine endometrial tissue explants on days 10 to 11, 12 to 13, 15 to 16, and 27 to 28 of the pregnancy, and on days 10 to 11 of the estrous cycle. The gene expression was determined by quantitative real-time PCR. The proteins secretion was determined by an ELISA test. Results are reported as the means ± S.E.M. (*n* = 5). Bars with different superscripts differ (*p* < 0.05).

In the endometrium, on days 10 to 11 of pregnancy, PGF_2α_ caused an increase (250, 500 ng/mL) in chemerin gene expression (Figure 5A). On days 12 to 13 of pregnancy, PGF_2α_ caused an increase in chemerin mRNA content (100, 250, 500 ng/mL), but decreased (500 ng/mL) its protein secretion (Figure 5C,D). On days 15 to 16 of gestation, PGF_2α_ caused a decrease in chemerin gene expression (100, 500 ng/mL), as well as its protein secretion (100, 250, 500 ng/mL; Figure 5E,F). On days 27 to 28 of pregnancy, PGF_2α_ also decreased both chemerin gene expression (100, 250, 500 ng/mL) and protein secretion (250 ng/mL) (Figure 5G,H; *p* < 0.05).

Briefly, PGF_2α_ decreased chemerin mRNA content on days 15 to 16 and 27 to 28 of pregnancy, and protein secretion on days 12 to 13, 15 to 16, and 27 to 28 of pregnancy. On days 10 to 11 and 12 to 13 of gestation, the prostaglandin enhanced chemerin gene expression.

### 2.6. The Effect of PGF_2α_ on CMKLR1 Gene and Protein Expression in the Endometrial Tissue Explants

**Figure 6:**
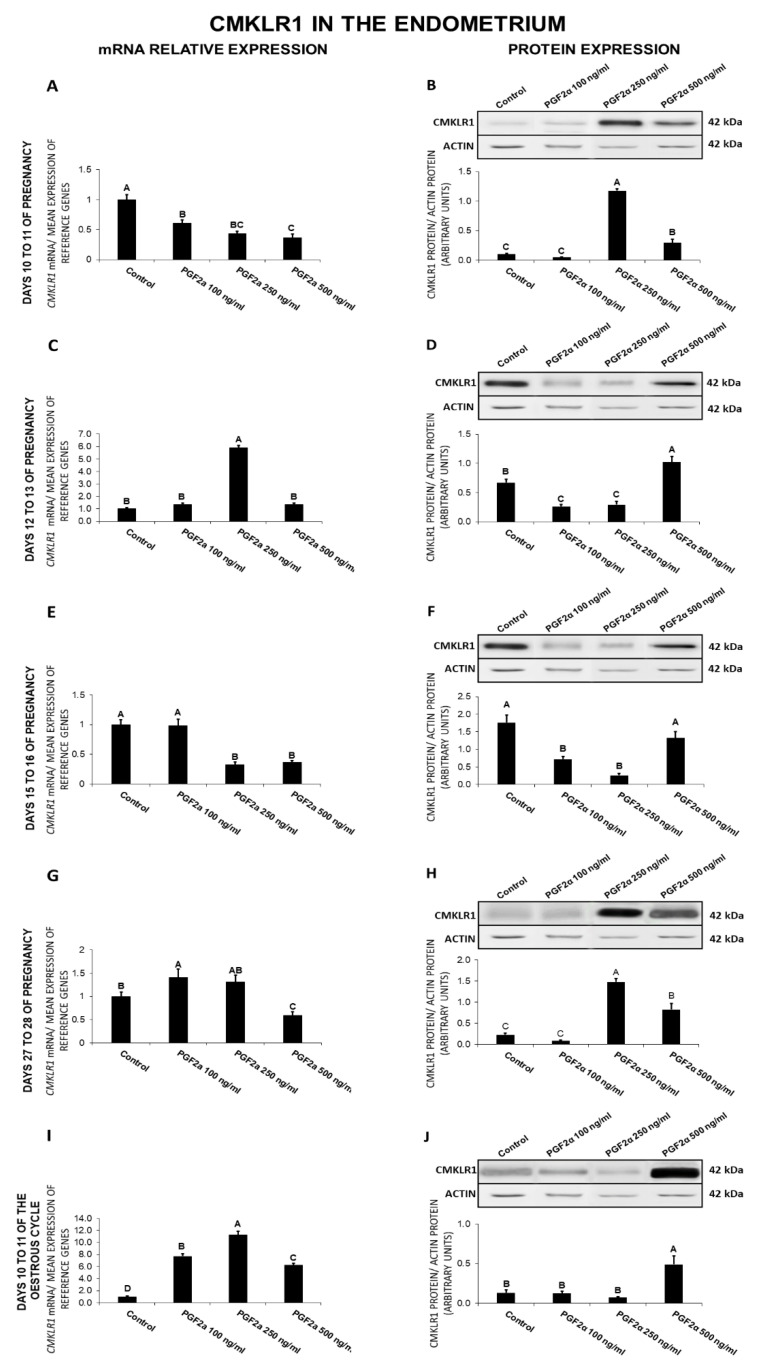
The influence of prostaglandin F_2α_ (PGF_2α_; 100, 250, 500 ng/mL) on chemokine-like receptor 1 (CMKLR1) mRNA (**A**,**C**,**E**,**G**,**I**) and protein (**B**,**D**,**F**,**H**,**J**) expression in the porcine endometrium on days 10 to 11, 12 to 13, 15 to 16, and 27 to 28 of the pregnancy, and on days 10 to 11 of the estrous cycle. The gene expression was determined by quantitative real-time PCR. The protein concentration was determined by the western blotting analysis; upper panels: Representative immunoblots; lower panels: Densitometric analysis of CMKLR1 protein relative to actin protein. Results are reported as the means ± S.E.M. (*n* = 5). Bars with different superscripts differ (*p* < 0.05).

On days 10 to 11 of gestation, PGF_2α_ caused a decrease (100, 250, 500 ng/mL) in *CMKLR1* expression and increased (250, 500 ng/mL) the receptor protein concentration (Figure 6A,B). On days 12 to 13 of pregnancy, PGF_2α_ at the dose of 250 ng/mL enhanced *CMKLR1* expression (Figure 6C). On those days, PGF_2α_ at the dose of 500 ng/mL increased, but at the doses of 100 and 250 ng/mL, decreased the receptor protein concentration (Figure 6D). On days 15 to 16 of pregnancy, PGF_2α_ caused a decrease in CMKLR1 expression at both the gene (250, 500 ng/mL) and protein (100, 250 ng/mL) levels (Figure 6E,F). On days 27 to 28 of pregnancy, PGF_2α_ at the dose of 100 ng/mL enhanced, but at the dose of 500 ng/mL inhibited *CMKLR1* expression (Figure 6G). On those days, PGF_2α_ at the doses of 250 and 500 ng/mL was found to enhance the receptor protein expression (Figure 6H). On days 10 to 11 of the estrous cycle, PGF_2α_ provoked an increase in both CMKLR1 gene (100, 250, 500 ng/mL) and protein (500 ng/mL) expression (Figure 6I,J; *p* < 0.05).

Summarizing, PGF_2α_ suppressed *CMKLR*1 gene expression on days 10 to 11 and 15 to 16 of pregnancy, and the receptor protein expression on days 15 to 16 of pregnancy. On days 12 to 13 of gestation and 10 to 11 of the cycle, PGF_2α_ enhanced the receptor gene expression, whereas on days 10 to 11, 27 to 28 of pregnancy and 10 to 11 of the cycle, it increased CMKlR1 protein expression. 

### 2.7. The Effect of PGF_2α_ on GPR1 Gene and Protein Expression in the Endometrial Tissue Explants

**Figure 7:**
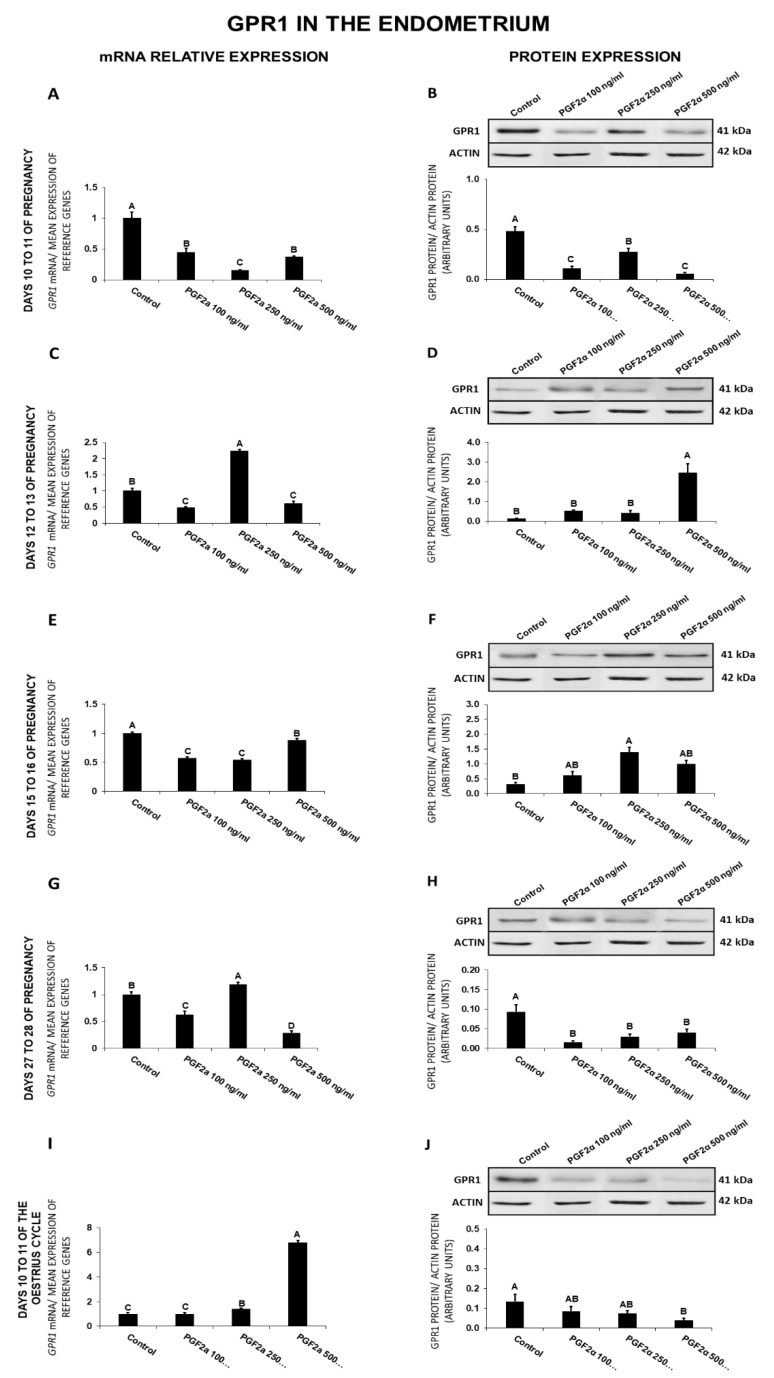
The influence of prostaglandin F_2α_ (PGF_2α_; 100, 250, 500 ng/mL) on G protein-coupled receptor 1 (GPR1) mRNA (**A**,**C**,**E**,**G**,**I**) and protein (**B**,**D**,**F**,**H**,**J**) expression in the porcine endometrium on days 10 to 11, 12 to 13, 15 to 16, and 27 to 28 of the pregnancy, and on days 10 to 11 of the estrous cycle. The gene expression was determined by quantitative real-time PCR. The protein concentration was determined by the western blotting analysis; upper panels: Representative immunoblots; lower panels: Densitometric analysis of GPR1 protein relative to actin protein. Results are reported as the means ± S.E.M. (*n* = 5). Bars with different superscripts differ (*p* < 0.05).

In the endometrium, on days 10 to 11 of gestation, PGF_2α_ at all the tested doses caused a decrease in GPR1 expression at both gene and protein levels (Figure 7A,B). On days 12 to 13 of gestation, PGF_2α_ at the dose of 250 ng/mL increased, whereas at the doses of 100 and 500 ng/mL decreased *GPR1* expression (Figure 7C). On those days, PGF_2α_ at the dose of 500 ng/mL enhanced the receptor protein expression (Figure 7D). On days 15 to 16 of pregnancy, PGF_2α_ caused a decrease (100, 250, 500 ng/mL) in *GPR1* expression but increased (250 ng/mL) the endometrial receptor protein concentration (Figure 7E,F). On days 27 to 28 of pregnancy, PGF_2α_ at the dose of 250 ng/mL increased, whereas at the doses of 100 and 500 ng/mL decreased the *GPR1* mRNA content (Figure 7G). On those days, PGF_2α_ at all the tested doses decreased the receptor protein concentration (Figure 7H). On days 10 to 11 of the estrous cycle, PGF_2α_ enhanced (250, 500 ng/mL) GPR1 expression and decreased (500 ng/mL) the receptor protein concentration (Figure 7I,J; *p* < 0.05).

Generally, PGF_2α_ decreased *GPR1* gene expression on days 10 to 11 and 15 to 16 of pregnancy and the receptor protein content on days 10 to 11 and 27 to 28 of gestation and 10 to 11 of the cycle. On days 10 to 11 of the cycle PGF_2α_ increased *GPR1* gene expression, and on days 12 to 13 and 15 to 16 of pregnancy, it increased the receptor protein content. 

### 2.8. The Effect of PGF_2α_ on CCRL2 Gene and Protein Expression in the Endometrial Tissue Explants

**Figure 8:**
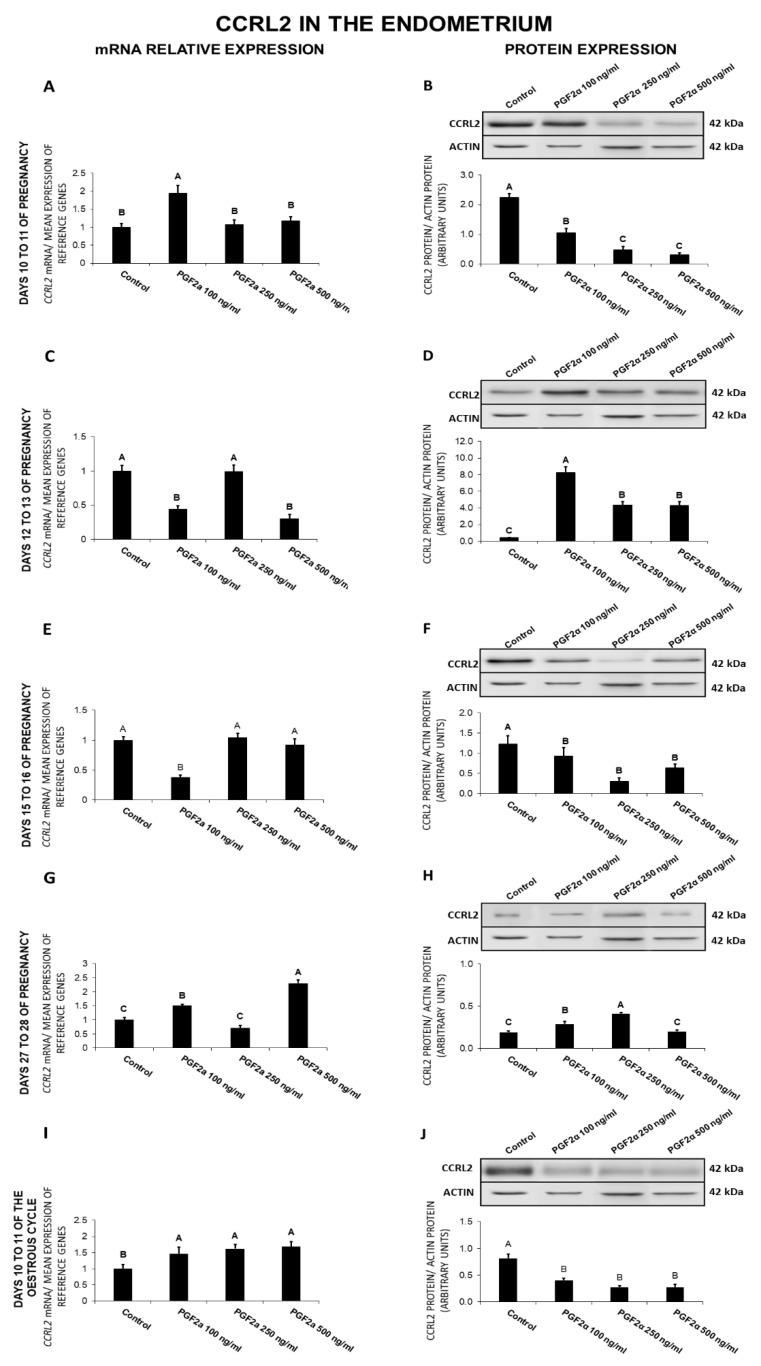
The influence of prostaglandin F_2α_ (PGF_2α_; 100, 250, 500 ng/mL) on C-C motif chemokine receptor like 2 (CCRL2) mRNA (**A**,**C**,**E**,**G**,**I**) and protein (**B**,**D**,**F**,**H**,**J**) expression in the porcine endometrium on days 10 to 11, 12 to 13, 15 to 16, and 27 to 28 of the pregnancy, and on days 10 to 11 of the estrous cycle. The gene expression was determined by quantitative real-time PCR. The protein concentration was determined by the western blotting analysis; upper panels: Representative immunoblots; lower panels: Densitometric analysis of CCRL2 protein relative to actin protein. Results are reported as the means ± S.E.M. (*n* = 5). Bars with different superscripts differ (*p* < 0.05).

In the endometrium, on days 10 to 11 of gestation, PGF_2α_ enhanced (100 ng/mL) *CCRL2* expression and decreased (100, 250, 500 ng/mL) the receptor protein concentration (Figure 8A,B). On days 12 to 13 of pregnancy, PGF_2α_ decreased (100, 500 ng/mL) *CCRL2* expression and increased (100, 250, 500 ng/mL) the receptor protein concentration (Figure 8C,D). On days 15 to 16 of pregnancy, PGF_2α_ caused a decrease in CCRL2 expression at both the gene (100 ng/mL) and protein (100, 250, 500 ng/mL) levels (Figure 8E,F). On days 27 to 28 of gestation, PGF_2α_ provoked an increase in *CCRL2* expression (100, 500 ng/mL), as well as in the receptor protein concentration (100, 250 ng/mL; Figure 8G,H). On days 10 to 11 of the estrous cycle, PGF_2α_ at all tested doses stimulated *CCRL2* expression, but decreased the receptor protein concentration (Figure 8I,J; *p* < 0.05).

In general, PGF_2α_ caused a decrease in *CCRL2* gene expression on days 12 to 13 and 15 to 16 of gestation, and in the receptor protein content on days 10 to 11 and 15 to 16 of gestation and 10 to 11 of the cycle. On days 10 to 11 and 27 to 28 of pregnancy and 10 to 11 of the cycle, PGF_2α_ enhanced *CCRL2* gene expression, whereas on days 12 to 13 and 27 to 28 of pregnancy, it increased the receptor protein content.

## 3. Discussion

The expression of the chemerin system in the porcine reproductive tract during the estrous cycle and early gestation has been reported previously [23]. Chemerin, CMKLR1, GPR1, and CCRL2 have been detected in both the endometrium and the myometrium. However, the relationship between the chemerin system and uterine-derived factors responsible for the regulation of reproductive functions, such as steroid hormones and prostaglandins, has not been previously investigated. This is the first study to demonstrate the modulatory influence of PGE_2_ and PGF_2α_ on chemerin system expression in the endometrium of early-pregnant gilts. The obtained results indicate the dose-dependent influence of prostaglandins (PGs) on the endometrial expression of chemerin system at both the gene and protein levels. Our unpublished results (Smolinska et al., unpublished) indicate that chemerin may also enhance the expression of the key enzymes responsible for the synthesis of prostaglandins such as microsomal prostaglandin E synthase-1 (mPGES-1), cyclooxygenase-2 (COX-2), prostaglandin F synthase (PGFS), and prostaglandin E 9-keto-reductase (CBR1), as well as modulate the secretion of PGE_2_ and PGF_2α_, depending on the adipokine dose and the pregnancy period. The above points to the presence of a complex regulatory loop between chemerin and prostaglandins in the porcine endometrium. This study revealed differences in the expression patterns of chemerin system genes and the corresponding proteins. The observed differences in the expression patterns of mRNA and the corresponding proteins are not surprising. Schwanhäusser et al. [27] reported that the differences in mRNA expression in mammals explained approximately 40% of the variation in protein levels, and the coefficient of determination (R^2^) between the content of mRNA and protein was only 0.41. The R^2^ value increased to 0.95 when translation rate constants were taken into account. These suggest that the protein abundance may be regulated mainly by the translation efficiency. The presented mechanism may be beneficial for the metabolism of cell because of the savings in the substrates for protein synthesis and the minimization in the energy expenditure associated with the translation process. The discrepancies between gene and protein expression may also arise from the differences in the stability of proteins and gene transcripts, transcriptional and post-transcriptional regulation, inhibition of post-transcriptional processes through high mRNA content, or attenuation of gene expression due to high protein concentrations, as well as due to miRNA-induced mRNA degradation [28,29,30]. The observed changes in the expression of the endometrial chemerin system under the influence of prostaglandins could be explained by variations in the expression of prostaglandin receptors during the analyzed pregnancy periods. The concentrations of PGE_2_ and PGF_2α_ receptors fluctuate during pregnancy in mice and during the estrous cycle and early gestation in pigs [31,32,33]. In pigs, differences were observed in the endometrial content of PGF_2α_ receptor (PTGFR) protein during the estrous cycle and gestation [32]. Estradiol and PGE_2_, the main factors responsible for the maternal recognition of pregnancy, stimulate the endometrial expression of PGE_2_ receptors PTGER2 and PTGER4 [33]. Varied responses to prostaglandins may also be explained by differences in the activity of prostaglandin 9-ketoreductase (CBR1) in explants collected during different gestation periods. The prostaglandin 9-ketoreductase enzyme converts PGE_2_ into PGF_2α_. The expression of the CBR1 gene and protein was confirmed in the porcine uterus during early pregnancy and the oestrous cycle [34], which could point to the conversion of prostaglandins in tissue explants during the studied periods. Therefore, the differences in the concentration of chemerin system elements may be explained by the characteristic response for each of the studied dose of PGs. Beside the concentration and ratio of both prostaglandins, their receptors expression patterns, which are strongly connected with the individual periods of early gestation, should also be considered as an important factor that may affect chemerin and its receptors’ mRNA and protein content. The differences observed in the present study could also be attributed to changes in the hormonal microenvironment induced by different concentration patterns of steroid hormones, endogenous prostaglandins, as well as the occurrence of accompanying processes characteristic for the studied periods.

Early pregnancy in pigs is one of the most dynamic and critical periods during gestation. Around day 11 of porcine gestation, migrating conceptuses begin to secrete E_2_, which initiates a number of processes related to the maternal recognition of pregnancy [35]. During this time, the direction of PGF_2α_ secretion is altered from endocrine to exocrine, and PGE_2_ production is stimulated, which promotes the secretory functions of the corpus luteum and ensures the proper course of gestation [36,37]. During our previous study we indicated the changes of chemerin system protein expression across early gestation which may be caused by a number of factors present in the uterine microenvironment [23]. The analysis of the results presented herein indicates many similarities between the basal and PG-stimulated chemerin system expression patterns on the corresponding days of gestation. The comparison of the response patterns indicates that changes in the basal chemerin system expression during early pregnancy seem to be connected with the local presence of PGE_2_ and PGF_2α_. Furthermore, due to a fact that PGs are secreted in a pulsatile manner, and their secretion patterns during early pregnancy are similar, the PGE_2_:PGF_2α_ ratio is another agent, which should be taken into consideration [38]. Beside its luteoprotective effects, the redirection of prostaglandin secretion also promotes endometrial reconstruction and modulation of the angiogenesis process during the peri-implantation period [32,39,40]. The chemerin system seems to be involved in the stimulation of angiogenesis. Chemerin has been found to promote angiogenesis in vivo in mice and in vitro in the human umbilical vein endothelial cell line. Similar effects were observed in the human microvascular endothelial cell line [41,42]. Furthermore, it has also been found that chemerin, besides its influence on the vascularization process, may also, mainly via CMKLR1 receptor, influence the vascular tone and blood pressure [43]. Our unpublished data also indicate that chemerin enhances the endometrial secretion of several angiogenic factors, including vascular endothelial growth factors (VEGFA and VEGFB), placental growth factor, and basic fibroblast growth factor, as well as modulates the expression of their receptor proteins, depending on chemerin dose and the stage of gestation. In the present study, PGE_2_ decreased CMKLR1 protein concentrations on days 12 to 13 and 15 to 16 of gestation, whereas increased CCRL2 protein expression. PGF_2α_ generally exerts a similar effect on the CMKLR1 receptor on days 12 to 16 of gestation and enhances CCRL2 protein content on days 12 to 13. Both prostaglandins exerted the opposite effects on GPR1 protein expression on days 12 to 16 of gestation. Chemerin promotes angiogenesis mainly via the CMKLR1 receptor [41], whereas the GPR1 receptor is responsible mainly for glucose homeostasis [44], which could suggest that by suppressing CMKLR1 and promoting GPR1 expression, prostaglandins could regulate tissue remodeling, prevent excessive vascular development, and ensure the availability of nutrients for implanting embryos. These findings could be confirmed by the observation that the lack of GPR1 expression in pregnant mice resulted in glucose intolerance, disrupted lipid metabolism, and decreased insulin levels [45]. We propose that the regulation of these processes may take place not only through the inhibition of CMKLR1 receptor expression, but also via the stimulation of CCRL2 protein expression. An increase of CCRL2 protein content may result in an increased chemerin uptake from the circulating blood and, in consequence, enhanced GPR1 receptor expression in the tissue. Interestingly, both prostaglandins stimulated the expression of CMKLR1 and CCRL2 proteins on days 27 to 28 of gestation, which implies that the chemerin system could be involved in intensive angiogenesis processes during this period. The present results suggest that the chemerin system and prostaglandins could be a part of a mechanism that regulates endometrial tissue vascularization and, consequently, nutrient availability.

In mammals, gestation is a unique period during which the maternal organism accepts conceptuses carrying different genetic material. Developing conceptuses harbor only one half of genetic material which is identical to the maternal DNA, and they are perceived as natural semi-allografts. The presence of foreign genetic material in the uterine environment raises the risk of immune system mobilization and, consequently, embryo rejection. The suppression of immune activity in the pregnant uterus is a natural mechanism that prevents embryo rejection. Implanting conceptuses secrete a number of factors that modulate the maternal immune system, including interleukin 1β (IL1β), interferons δ and γ, interleukin 6 (IL6), epidermal growth factor, transforming growth factor β, and leukemia inhibitory factor (LIF), which are crucial for maternal–fetal crosstalk [46,47,48,49,50]. However, these processes must be strictly controlled to prevent conceptus rejection [51,52]. Progesterone and PGE_2_ are the key factors that suppress the immune system during gestation. The presence of PGE_2_ during the first trimester of human gestation restrains the activation of maternal leukocytes in the decidua, which inhibits their anti-trophoblast killer function [53]. In contrast, PGF_2α_ is an important inflammatory mediator in the reproductive system [54]. Chemerin is also a potential modulator of the immune response. Chemerin acts through CMKLR1 to promote chemotaxis of immature dendritic cells (DCs) and macrophages [6]. Chemerin levels were elevated in tissues and fluids during inflammation, and CMKLR1-expressing immune cells were involved in several chronic inflammatory diseases [5]. However, chemerin can also exert anti-inflammatory effects. Cash et al. (2008) reported that chemerin significantly downgraded neutrophil and monocyte recruitment and decreased proinflammatory cytokine expression in mice [55]. Our unpublished data indicate that chemerin may modulate the endometrial secretion of various cytokines, including interleukins 1β, IL6 and 8, tumor necrosis factor α (TNFα), transforming growth factor α (TGFα), and LIF. We revealed that chemerin may inhibit and/or enhance the secretion of IL8, IL1β, IL6, TNFα, TGFα, and LIF in a dose- and time-dependent manner. Chemerin not only influences cytokine secretion, but also controls cytokine activity by regulating the expression of cytokine receptors in endometrial tissue. We found that the effect of chemerin on the cytokine receptor expression in the endometrium varied depending on the pregnancy period and the dose of the adipokine (Smolinska et al., unpublished). Due to the fact that chemerin acts as the chemoattractant for the immune cells, one of the most important sources of cytokines, further studies concerning the role of this adipokine in the endometrial immune cells recruitment are necessary to receive the full picture of its effect on the uterine immunological milieu during the early gestation period. The current study revealed that PGE_2_ inhibits chemerin secretion on days 15 to 16 of pregnancy and stimulates CCRL2 protein expression, whereas PGF_2α_ exerts the same effect on days 12 to 28 of gestation in the case of chemerin and promotes the expression of CCRL2 on days 12 to 13 and 27 to 28. During implantation, on days 15 to 16 of pregnancy, both prostaglandins could inhibit chemerin production to prevent the recruitment of immune cells and pregnancy failure. Furthermore, the enhanced expression of CCRL2 protein in the tissue may result in further chemerin level reduction. In pigs, the conceptuses initiate an acute-phase inflammatory response during implantation and placental attachment; therefore, PGF_2α_ could inhibit chemerin secretion during the maternal recognition of pregnancy and implantation to counteract its anti-inflammatory effects and establish a supportive environment for gestation [56].

## 4. Materials and Methods

### 4.1. Animals and Tissue Collection

**Figure 9:**
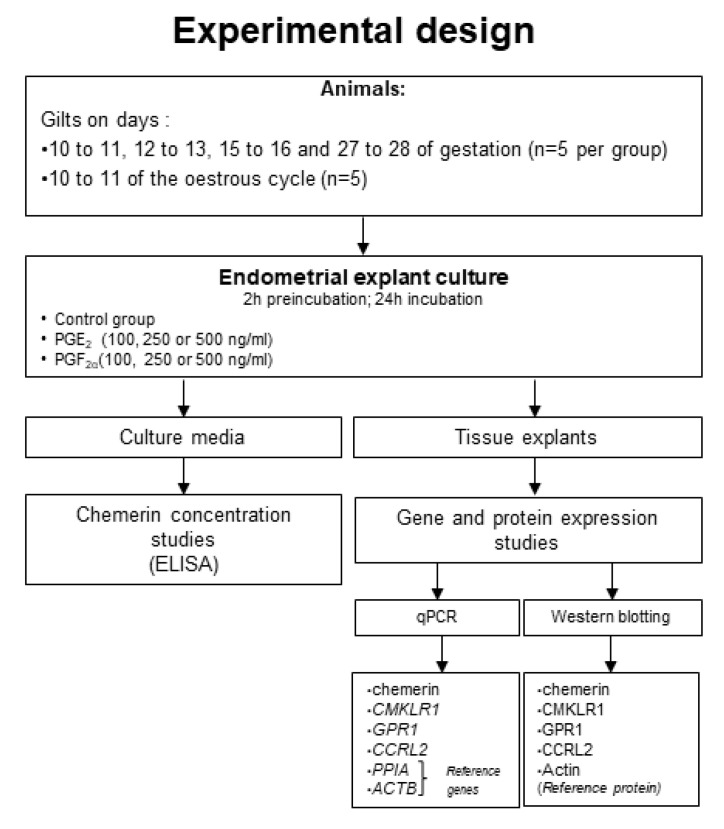
Experimental design graph.

Twenty-five mature crossbred gilts (Large White × Polish Landrace, 130–140 kg, of weight and age of 7–8 months) were randomly assigned to one of the following experimental groups (n = 5 per group): Gilts on days 10 to 11 (transuterine migration of embryos), 12 to 13 (maternal recognition of pregnancy), 15 to 16 (beginning of implantation), and 27 to 28 (end of implantation) of pregnancy, and days 10 to 11 of the estrous cycle (mid-luteal phase; the activity of corpora lutea (CL) is comparable to the CL activity during pregnancy). The quantity of CLs and embryos are specified in the Supplementary Table S1. Animals were maintained and fed in accordance with current Polish standards. The daily dose of compound feed was approximately 2.7 kg/gilt at 32.4 MJ of metabolizable energy intake (12 MJ per kg of feed). The feed contained 135 g/kg of total digestible protein, 5.4 g/kg methionine and cystine, 7.3 g/kg lysine, 2.1 g/kg tryptophan, 4.8 g/kg threonine, 5.7 g/kg total phosphorus, 8.9 g/kg calcium, 1.7 g/kg sodium, 10% fiber, and the addition of other macro- and microelements. All individuals were given access to fresh water and forage *ad libitum*. Cyclic gilts were monitored daily for estrus behavior in the presence of boar. The day of the onset of the second estrus was recognized as day 0 of the cycle. The phase of the estrus cycle was confirmed on the basis of the ovary morphology [57]. The inseminations were conducted by natural mating with the use of the same, crossbreed boar. The insemination was carried out on the first or second day of the estrous cycle, and the first day after coitus was counted as a first day of gestation. The phase of gestation was further confirmed based on the conceptuses morphology [58]. Additionally, the phase of the estrous cycle and pregnancy was confirmed by determining the level P_4_, as described in Nitkiewicz et al. [59] and Dobrzyn et al. [60]. The concentrations of P_4_ in the porcine plasma and uterine luminal flushing are detailed in the Supplementary Table S1. The post-mortem-obtained uteri were transported immediately in ice-cold PBS supplemented with antibiotic-antimycotic solution (Sigma-Aldrich, Saint Louis, MO, USA) for an in vitro tissue culture procedure. Figure 9 presents the experimental design scheme.

### 4.2. Endometrial Explant Cultures

The in vitro cultures of endometrial explants were conducted as described by Smolinska et al. [61]. In order to investigate the impact of PGE_2_ and PGF_2α_ on the chemerin system expression, after preincubation (2 h) in phenol-red free medium M199 (Sigma-Aldrich, USA), explants were incubated in the presence of PGE_2_ (100, 250, 500 ng/mL; Sigma-Aldrich, USA), PGF_2α_ (100, 250, 500 ng/mL; Sigma-Aldrich, USA), or without any treatment (control group) for 24 h (37 °C, 95% O_2_, 5% CO_2_). The doses of PGE_2_ and PGF_2α_ were chosen based on the work of Morgan et al. [62] and Gregoraszczuk and Michas [63]. Morgan et al. measured PGF_2α_ levels in porcine uterine flushing on day 11 of gestation under exposure to estradiol valerate. The concentration of PGF_2α_ in the control group was 198 ng/mL on the above-mentioned day. Since both PGs are secreted in a pulsatile manner and their concentrations are not constant, their physiological levels could be determined in the range of 100–500 ng/mL. The cultures were run in five separate experiments per group (n = 5, one gilt per each experiment) and each treatment combination was prepared in duplicates. The lactate dehydrogenase (LDH) activity measurement after preincubation and incubation periods was used to define the viability of tissue explants. The analysis was performed using Liquick Cor-LDH kit (Cormay, Poland) in accordance with the manufacturer’s instructions. The obtained LDH activity in the culture media was compared to the enzyme activity in the fully disintegrated tissue (maximal LDH release, positive control). The mean activity of LDH in the culture media after 24 h of incubation was 184 ± 14 U/L (0.93% of maximal LDH release).

### 4.3. Total RNA Isolation and Reverse Transcription

Total RNA from endometrial tissue explants was isolated using TRI Reagent^®^ RNA Isolation Reagent (Sigma-Aldrich) following the producer’s instructions. The quantity and purity of the obtained RNA samples were inspected spectrophotometrically using Infinite M200 Pro (Tecan, Mannedorf, Switzerland). One microgram of each RNA sample was reverse transcribed (RT) with the use of Omniscript RT Kit (Qiagen, Germantown, MD, USA) in the presence of 0.5 μg oligo(dT)_15_ (Roche, Basel, Switzerland) in a final volume of 20 μL. The RT reaction was conducted at 37 °C for 1 h and was terminated by the incubation at 93 °C for 5 min.

### 4.4. Quantitative Real-Time PCR Analysis

Quantitative real-time PCR (qPCR) analysis was carried out using Power SYBR Green Master Mix (Applied Biosystems, Carlsbad, CA, USA) as described by Smolinska et al. [14]. Specific primer pairs used to amplify parts of chemerin, *CMKLR1, GPR1, CCRL2,* cyclophilin (*PPIA*), and β-actin (*ACTB*) genes are included in Table 1. The constitutively expressed genes *PPIA* and *ACTB* were used as the internal controls to verify the method. Our preliminary studies revealed that the endometrial *PPIA* and *ACTB* expression was stable during the estrous cycle and pregnancy, as well as with and without treatments. Quantitative real-time PCR reaction mixtures contained: cDNA, primers, Power SYBR Green PCR Master Mix (12.5 μL; Applied Biosystems), and RNase-free water (to the final volume of 25 μL). In negative controls, the cDNA was substituted by water, or RT was not performed before qPCR. All reactions were run in duplicates. The specificity of the reaction was confirmed at the end of the run by the analysis of the melting-curve. The purity of the amplification product was confirmed by agarose gel electrophoresis. The calculation of chemerin and chemerin receptors genes’ relative expression was conducted with the use of the comparative cycle threshold method (ΔΔCT) and normalized using the geometrical means of the reference genes Ct values.

### 4.5. Enzyme-Linked Immunosorbent Assay (ELISA) of Chemerin

Chemerin concentrations in the culture media were determined using a commercial ELISA kit (FineTest, Wuhan, China) following the manufacturer’s protocol. The range of standard curve was 0.156–10 ng/mL. The sensitivity of the assay was approximately 0.1 ng/mL. The sensitivity of the assays was defined as the lowest protein concentration that could be differentiated from zero samples. Absorbance was measured at 450 nm with the use of Infinite M200 PRO reader with Tecan i-control software (Tecan, Switzerland). The data were linearized by plotting the log of chemerin concentration versus the log of the optical density, and the best fit line was determined by regression analysis. Intra- and inter-assay coefficients of variation of the assay were 3.22% ± 0.39 and 8.1%, respectively.

The basal concentrations of chemerin in the uterine luminal flushing were determined by [23] and are detailed in the Supplementary Table S1.

### 4.6. Protein Isolation and Western Blotting

The endometrial tissue explants were homogenized in T-PER Tissue Protein Extraction Reagent (Thermo Fischer Scientific, Waltham, MA, USA) in the presence of peptidase and phosphatase inhibitors (Sigma-Aldrich). The lysates were cleared by double centrifugation at 10,000× *g* for 5 min. The protein concentrations were measured with the use of Bradford dye-binding procedure with the dilutions of bovine serum albumin (BSA) as standards.

Western blotting analysis was performed as described by Smolinska et al. [14]. Endometrial tissue lysates (40 μg) from control, PGE_2_-, and PGF_2α_-treated samples were resolved by SDS-PAGE electrophoresis in the 12.5% polyacrylamide gels and transferred onto PVDF membrane (Whatman, USA). Subsequently, membranes were blocked for 1 h in Tris-buffered saline Tween-20 containing 5% skimmed milk powder. After blocking, membranes were incubated for 12 h at 4 °C with rabbit polyclonal antibodies to CMKLR1 (1:1000; ab230442; Abcam, UK), mouse polyclonal antibodies to GPR1 (1:500; ab169331; Abcam, UK), rabbit polyclonal antibodies to CCRL2 (1:600; ab85224; Abcam, UK), and rabbit polyclonal antibodies to actin (1:200; A2066; Sigma-Aldrich, USA). Actin was used as a control to normalize the results of chemerin receptors protein concentration. Subsequently, to identify immunoreactive products, membranes were incubated for 1.5 h at RT with goat anti-rabbit IgG for CMKLR1, CCRL2, and actin (1:5000; sc-2054; Santa Cruz, USA), and goat anti-mouse IgG for GPR1 (1:2500; 115-035-003; Jackson ImmunoResearch Laboratories, Baltimore Pike, PA, USA) conjugated with horseradish peroxidase (HRP). For negative control blots, primary antibodies were substituted by nonspecific fetal calf serum (MP Biomedicals, Santa Ana, CA, USA). Immunocomplexes were visualized with Immobilon Western Chemiluminescent HRP Substrate (Merck Millipore, Kenilworth, NJ, USA) on the G: Box EF Gel Documentation System (Syngene, Cambridge, UK). The same protocol was performed in relation to the adipose tissue used as the positive controls. The results were quantified by densitometric analysis of immunoblots with the use of Image Studio Lite version 5.2 software (LI-COR, Lincoln, NE, USA). Data were expressed as the ratio of chemerin receptors proteins relative to actin protein in arbitrary optical density units.

### 4.7. Statistical Analysis

Statistica software (StatSoft Inc., Tulsa, OK, USA) was used to perform the statistical analysis of results. All variables were analyzed using descriptive statistics (mean, standard deviation, sample minimum, and sample maximum). To determine the differences in genes expression and proteins concentration between control groups and PGE_2_ or PGF_2α_ treated groups, a one-way ANOVA followed by Duncan’s post-hoc test were used. Results were presented as the means ± S.E.M. from five independent observations. Values for *p* < 0.05 were considered statistically significant.

## 5. Conclusions

This is the first study to demonstrate that prostaglandins affect the expression of the chemerin system in the porcine uterus during early gestation. The presented results indicate that the analyzed prostaglandins exert different effects on the endometrial chemerin system expression, which depend on the dose of the used PGs. Furthermore, we revealed that the response pattern for PGs is specific for each of the studied pregnancy periods. The above findings confirm our hypothesis assuming the regulatory influence of PGs on chemerin system expression in the porcine early-pregnant uterus. In the light of the existing knowledge, the presented findings also suggest that the chemerin system could be an important element of the regulatory mechanism responsible for the proper course of gestation, and that its expression depends on the local hormonal microenvironment.

## Figures and Tables

**Table 1:** Characteristics of the primers used in the study of gene expression in porcine endometrial explants. *RARRES2*: Chemerin; *CCRL2*: C-C motif chemokine receptor like 2; *CMLKR1*: chemokine-like receptor 1; *GPR1*: G protein-coupled receptor 1; *PPIA*: Cyclophilin; *ACTB*: β-actin; F: Forward; R: Reverse.

Gene	Primers Sequences	Accession Number	Primer, nM	Reaction Conditions		Reference
*RARRES2*	F: 5′-TGGAGGAGTTCCACAAGCAC-3′	EU660865	500	$\begin{array}{c} \mathrm{Activation}:\;95\;{}^{\circ}C—10\;\min\\ \begin{array}{c} \mathrm{Denaturation}:\;95\;{}^{\circ}C—15\;s\\ \mathrm{Annealing}:\;60\;{}^{\circ}C—1\;\min\\ \mathrm{Elongation}:\;72\;{}^{\circ}C—1\;\min \end{array}\} \end{array}$	40	[14]
	R: 5′-GCTTTCTTCCAGTCCCTCTTC-3′		500			
*CCRL2*	F: 5′-GAGCAGCAGCTACTTACTTCC-3′	NM_001001617.1	200	$\begin{array}{c} \mathrm{Activation}:\;95\;{}^{\circ}C—10\;\min\\ \begin{array}{c} \mathrm{Denaturation}:\;95\;{}^{\circ}C—15\;s\\ \mathrm{Annealing}:\;60\;{}^{\circ}C—1\;\min\\ \mathrm{Elongation}:\;72\;{}^{\circ}C—1\;\min \end{array}\} \end{array}$	40	[14]
	R: 5′-CTGCCCACTGACCGAGTTC-3′		200			
*CMKLR1*	F: 5′-GGACTACCACTGGGTGTTCG-3′	EU660866	200	$\begin{array}{c} \mathrm{Activation}:\;95\;{}^{\circ}C—10\;\min\\ \begin{array}{c} \mathrm{Denaturation}:\;95\;{}^{\circ}C—15\;s\\ \mathrm{Annealing}:\;60\;{}^{\circ}C—1\;\min\\ \mathrm{Elongation}:\;72\;{}^{\circ}C—1\;\min \end{array}\} \end{array}$	40	[14]
	R: 5′-GCCATGTAAGCCAGTCGGA-3′		200			
*GPR1*	F: 5′-ACCGACTTGGAGGAGAAAGC -3’	FJ234899.1	200	$\begin{array}{c} \mathrm{Activation}:\;95\;{}^{\circ}C—10\;\min\\ \begin{array}{c} \mathrm{Denaturation}:\;95\;{}^{\circ}C—15\;s\\ \mathrm{Annealing}:\;60\;{}^{\circ}C—1\;\min\\ \mathrm{Elongation}:\;72\;{}^{\circ}C—1\;\min \end{array}\} \end{array}$	40	[14]
	R: 5′-ATTGAGGAACCAGAGCGTGG -3’		200			
*PPIA*	F: 5′-GCACTGGTGGCAAGTCCAT-3’	U48832	300	$\begin{array}{c} 50\;{}^{\circ}C—2\;\min\;{}^{\circ}C\\ \begin{array}{c} \mathrm{Activation}:\;95\;{}^{\circ}C—10\min\;{}^{\circ}C\\ \mathrm{Denaturation}:\;95\;{}^{\circ}C—15\;s\\ \mathrm{Annealing}:\;60\;{}^{\circ}C—1\;\min \end{array}\} \end{array}$	40	[64]
	R: 5′-AGGACCCGTATGCTTCAGGA-3’		300			
*ACTB*	F: 5′-ACATCAAGGAGAAGCTCTGCTACG-3’	U07786	500	$\begin{array}{c} \mathrm{Activation}:\;95\;{}^{\circ}C—10\;\min\\ \begin{array}{c} \mathrm{Denaturation}:\;95\;{}^{\circ}C—15\;s\\ \mathrm{Annealing}:\;61\;{}^{\circ}C—15\;\min\\ \mathrm{Elongation}:\;72\;{}^{\circ}C—1\;\min \end{array}\} \end{array}$	40	[65]
	R: 5′-GAGGGGCGATGATCTTGATCTTCA-3’		500			

## Supplementary Materials

Supplementary materials can be found at https://www.mdpi.com/1422-0067/21/15/5213/s1. Supplementary Table S1. Progesterone and chemerin concentrations, as well as and number of corpora lutea (CL) and embryos in the research groups. Supplementary Figure S1. The standard curve of Chemerin ELISA kit used in the study.

## Animal Welfare Statement

The authors confirm that the ethical policies of the journal, as noted on the journal’s author guidelines page, have been adhered to. All studies were carried out in accordance with the Polish Act on the protection of animals used for scientific or educational purposes of the 15th of January 2015 (Polish Journal of Law of 2015 item 266), as well as directive 2010/63/EU of the European Parliament of the 22nd of September 2010 on the protection of animals used for scientific purposes.
