# Supplementary material for: The In Vitro Effect of Prostaglandin E2 and F2α on the Chemerin System in the Porcine Endometrium during Gestation
Source: Int J Mol Sci. 2020 Jul 23;21(15):5213. doi: 10.3390/ijms21155213 (PMC7432131; doi:10.3390/ijms21155213)
Supplement: Supplementary file 1 [file ijms-21-05213-s001.pdf]

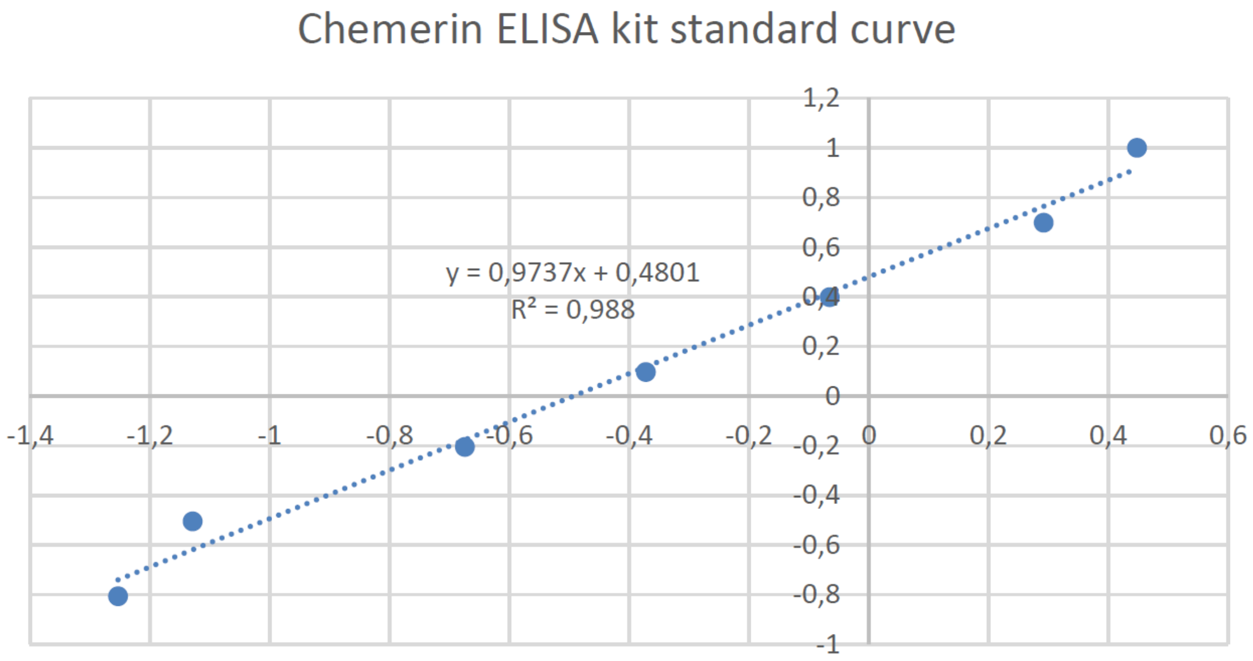

Supplementray Figure 1. Chemerin ELISA kit (FineTest, China) standard curve. The range of standard curve was 0.156–10 ng/mL. The sensitivity of the assay was approximately 0.1 ng/mL.

Table S1. Progesterone and chemerin concentrations, as well as number of corpora lutea (CL) and embryos in the research groups. Progesterone and chemerin concentrations has been reported as the means ± S.E.M. (n = 5).

Research group	Number of CLs/embryos	Progesterone concentration in plasma (ng/mL)	Progesterone concentration in uterine luminal flushing (ng/mL)	Chemerin concentration in uterine luminal flushing (ng/mL)
Days 10 to 11 of gestation	13–15 CLs	16.3 ± 0.7	0.3 ± 0.03	89.74 ± 14.53
Days 12 to 13 of gestation	13–15 CLs	14.7 ± 0.5	1.0 ± 0.2	460.25 ± 32.37
Days 15 to 16 of gestation	13–15 CLs	12.9 ± 0.9	1.0 ± 0.1	281.58 ± 43.24
Days 27 to 28 of gestation	13–20 CLs<br>10–14 embryos	11.7 ± 0.5	0.8 ± 0.03	7.72 ± 1.88
Days 10 to 11 of the cycle	13–15 CLs	19 ± 3.4	1.1 ± 0.3	96.29 ± 9.1
